# Intragraft donor-specific antibodies reflect histologic heterogeneity in kidney allografts with concurrent serum DSA

**DOI:** 10.3389/fimmu.2026.1878975

**Published:** 2026-06-23

**Authors:** Hyunjae Lee, Eun Youn Roh, Tae-Shin Kim, Yu Jung Choi, Inseong Oh, Hajeong Lee, Sang Il Min, Chang Wook Jeong, Hee Gyung Kang, Jongwon Ha, Kyung Chul Moon, Eun Young Song

**Affiliations:** 1 Department of Laboratory Medicine, Seoul National University College of Medicine, Seoul, Republic of Korea; 2 Department of Laboratory Medicine, Seoul Metropolitan Government - Seoul National University (SMG-SNU) Boramae Medical Center, Seoul, Republic of Korea; 3 Department of Laboratory Medicine, National Fire Hospital, Eumseong, Chungcheongbuk-do, Republic of Korea; 4 Department of Laboratory Medicine, Seoul National University Hospital, Seoul, Republic of Korea; 5 Department of Internal Medicine, Seoul National University Hospital, Seoul, Republic of Korea; 6 Department of Surgery, Seoul National University Hospital, Seoul, Republic of Korea; 7 Department of Urology, Seoul National University Hospital, Seoul, Republic of Korea; 8 Department of Pediatrics, Seoul National University Hospital, Seoul, Republic of Korea; 9 Department of Pathology, Seoul National University College of Medicine, Seoul, Republic of Korea

**Keywords:** antibody-mediated rejection, Banff classification, biopsy eluate, HLA antibodies, intragraft donor-specific antibodies, kidney transplantation, Luminex single antigen bead assay

## Abstract

**Background:**

Circulating donor-specific antibodies (sDSA) are clinically important markers of alloimmune risk, but their presence or strength does not always correspond to concurrent antibody-mediated rejection (ABMR) in kidney transplantation. We investigated whether intragraft donor-specific antibodies (gDSA) detected in kidney biopsy eluates are associated with histologic findings in recipients with concurrent sDSA positivity.

**Methods:**

All available frozen kidney biopsies in recipients with concurrent weak to moderate sDSA at Seoul National University Hospital between January 2020 and June 2024 were included. Sixty biopsy eluates from 60 patients were tested using a Luminex single antigen bead assay and analyzed according to biopsy findings.

**Results:**

The prevalence of gDSA differed significantly across histologic diagnoses (overall *P =* 3.3 × 10^-4^), with higher positivity in C4d-only lesions (100.0%), aABMR (90.9%) and aABMR+TCMR (83.3%), whereas lower frequencies were observed in caABMR (44.4%), no rejection (22.7%), and aTCMR (14.3%). The gDSA positive rate in aABMR was significantly higher than that in the no-rejection group (corrected *P =* 0.003; OR 34, 95% CI 3.5–334). The MFI sum of gDSA was significantly higher in aABMR, aABMR+TCMR, and C4d-only lesions (corrected *P =* 0.009, 0.035, and 0.007, respectively). In contrast, the sum of sDSA MFI did not differ significantly across the histologic groups.

**Conclusions:**

In kidney transplant recipients with concurrent sDSA, the presence and intensity of gDSA varied according to biopsy-proven histologic findings, most prominently in aABMR and related lesions. These findings suggest that gDSA may provide additional tissue-level immunologic context for understanding histologic heterogeneity among kidney transplant recipients with circulating sDSA.

## 1 Introduction

Donor-specific antibodies (DSA) to human leukocyte antigens (HLA) contribute significantly to the pathogenesis of antibody-mediated rejection (ABMR) in kidney transplantation (1). The interaction between DSA and intragraft vascular endothelium triggers immune responses that can lead to endothelial injury, inflammatory responses, and ultimately ABMR (2). Numerous studies have demonstrated that recipients with circulating DSA in serum (sDSA), whether preformed or *de novo*, are at increased risk of developing ABMR and graft failure compared to those without sDSA (3, 4). Therefore, various strategies have been implemented to prevent and detect the formation of sDSA. These approaches include high-resolution HLA typing (5), management of recipients with a history of sensitizing events (6) and use of sensitive assays for sDSA detection (7). The emergence of highly sensitive assays to detect sDSA such as Luminex-based platforms has enabled further understanding of how sDSA status before and after kidney transplantation impacts clinical outcomes (8). sDSA HLA specificities and strength, typically expressed as mean fluorescence intensity (MFI), are now widely used for risk stratification in kidney recipients (9).

However, the presence of circulating sDSA does not invariably lead to the development of ABMR in kidney recipients (10). In a study with 1,000 kidney recipients and five-year observation, the prevalence of acute ABMR among recipients with sDSA remained below 50% at all evaluated time points (11). Another study with 123 kidney recipients and more than 10-year observation demonstrated that over 40% of recipients with sDSA maintained long-term graft function (12).

We hypothesized that, among recipients with concurrent weak-to-moderate sDSA, gDSA may reflect local antibody–graft interaction not fully captured by circulating sDSA strength. To test this hypothesis, we analyzed gDSA in kidney biopsy eluates from recipients with concurrent weak to moderate sDSA across a spectrum of biopsy-proven histologic diagnoses and evaluated its association with histologic findings.

## 2 Materials and methods

### 2.1 Subjects and clinical data

Sixty frozen kidney biopsy tissues from 60 recipients who underwent biopsy between January 2020 and June 2024 at Seoul National University Hospital (SNUH) with concurrent weak to moderate levels of sDSA (MFI 500–10,000) by a single antigen bead assay were included (13). ABO-incompatible transplantation cases with abnormal biopsy findings were excluded to rule out tissue injury attributable to ABO antibodies. Graft biopsies were performed per protocol in 27 biopsies (45.0%) and for cause in 33 biopsies (55.0%). We retrospectively collected the following data: age, sex, relation to donor (living vs deceased), HLA typing, preformed sDSA status, pre-transplant desensitization therapy, maintenance immunosuppressive regimen at the time of biopsy, anti-rejection therapy administered before biopsy, single antigen bead assay results, C1q binding assay results, and kidney biopsy results during follow-up. The sDSA C1q binding assay was performed at the discretion of treating physicians and was available in 23 of the 60 recipients. The study was conducted following the Declaration of Helsinki and approved by the Institutional Review Board of Seoul National University Hospital (IRB No. 2309-105-1467).

### 2.2 Histopathology

Graft biopsies were retrospectively reviewed and categorized by two kidney pathologists at SNUH according to the Banff 2022 classification framework (14). Biopsies originally interpreted before the publication of the Banff 2022 update were re-categorized accordingly, with no resulting change in diagnostic category. Evaluation of C4d staining was performed on formalin-fixed, paraffin-embedded needle biopsy tissue by immunohistochemistry. The histologic findings were categorized into no rejection (n = 22), active antibody-mediated rejection (aABMR) (n = 11), C4d staining without evidence of rejection (C4d-only) (n = 3), acute T cell-mediated rejection (aTCMR) (n = 7), aABMR+TCMR (n = 6), chronic active ABMR (caABMR) ± interstitial fibrosis and tubular atrophy (IFTA) II (n = 9), and IFTA III (n = 2). Given the exploratory nature of this study and with the aim of minimizing selection bias, all consecutive biopsies meeting the inclusion criteria were included regardless of histologic subgroup size. This approach resulted in some categories with small numbers of biopsies, particularly C4d-only (n = 3) and IFTA III (n = 2), and findings from these subgroups should be interpreted with caution.

### 2.3 Intragraft antibody eluate

Intragraft antibody eluate was prepared in accordance with the previous literature (15–17). 1) Frozen kidney biopsy tissue was thawed at room temperature, and the tissue length was measured. The tissue was then minced in cold phosphate-buffered saline (PBS). 2) The minced kidney tissue suspended in cold PBS was transferred to a 1.5 mL microcentrifuge tube and centrifuged at 3, 000 rpm for 2 minutes. 3) The supernatant was discarded, and the pellet was resuspended in 1.5 mL of cold PBS. The suspension was then centrifuged at 3,000 rpm for 2 minutes. This washing step was repeated four times to remove residual serum from the graft. To confirm the completeness of the washing process, the final supernatant obtained after the last centrifugation was tested for HLA antibody and found to be negative. 4) Fifty microliters of the acid elution reagent, Diacidel (Bio-Rad, Hercules, CA, USA), was added to the tube, which was then incubated at room temperature for 10 minutes to elute graft-bound antibodies. 5) The tube was subsequently centrifuged at 6,000 rpm for 2 minutes, and the eluate was neutralized by adding 50 µL of 1 M Tris-HCl buffer (pH 8.5).

### 2.4 HLA antibody test and HLA genotyping

Detection of HLA antibodies was performed using the LABScreen Single Antigen HLA Class I/II assay (One Lambda, Canoga Park, CA, USA), according to the manufacturer’s instructions. 1) Twenty microliters of the eluate and 5 µL of the bead mixture were added to each well of a 96-well plate and incubated at room temperature for 30 minutes. 2) The plate was washed three times with phosphate-buffered saline (PBS). 3) One hundred microliters of phycoerythrin-conjugated anti-human IgG was then added to each well, followed by incubation at room temperature for 30 minutes. 4) After incubation, the beads were analyzed using a Luminex 200 system (Luminex Corporation, Austin, TX, USA), and the mean fluorescence intensity (MFI) of each HLA antibody was determined.

HLA genotyping was performed using LABType™ SSO (One Lambda, Canoga Park, CA, USA) for HLA-A, -B, -DRB1, -DQA1, and -DQB1. Additional typing for DRB3/4/5 (DR51/52/53) and high-resolution typing of HLA-A, -B, -DRB1, -DQA1, and -DQB1 were performed using NGSgo^®^ kits (GenDx, Utrecht, The Netherlands), when required for DSA identification.

Serum donor-specific antibodies (sDSA) were considered positive at MFI ≥500. For biopsy eluates, a lower positivity threshold of MFI ≥100 was applied. This cutoff was determined using a negative control matrix consisting of the acid elution reagent and neutralizing buffer used during the elution procedure. The negative control matrix was tested in 10 independent runs, and the biopsy-specific cutoff was defined as the mean plus 5 standard deviations of the negative control bead MFI values, corresponding to approximately 100. This threshold was also consistent with those used in previous gDSA studies (15, 16). As a complementary approach, a bead-based background assessment using donor-non-expressed allele beads was additionally performed as described by Courant et al. (17). This analysis yielded a background-derived MFI cutoff comparable to that obtained with the negative control matrix, further supporting the robustness of the applied threshold. Sensitivity analyses were additionally performed using alternative MFI cutoffs (≥50 and ≥150).

The sum of gDSA MFI values was used as the primary measure of gDSA strength; the highest gDSA MFI value was additionally analyzed as a secondary measure. To account for variability in biopsy tissue amount, the gDSA MFI sum was normalized to the cohort mean biopsy length, assuming a constant cross-sectional area of core biopsy specimens. The adjusted MFI sum was calculated as raw MFI sum × mean biopsy length/individual biopsy length.

### 2.5 Epitope analysis of graft eluate HLA antibodies

Epitope analysis was performed on biopsy eluates to determine whether eluted non-DSA anti-HLA antibodies shared epitopes with donor HLA antigens and could therefore be considered anti-donor HLA epitope-specific antibodies (18). The MFI sum of graft anti-donor HLA epitope-specific antibodies (gDESA) was calculated by adding the MFI value of all positive alleles sharing donor HLA epitope. The MFI sum of gDESA was also analyzed according to histologic groups.

### 2.6 Statistical analysis

The chi-square test or Fisher’s exact test was used to compare gDSA positivity between groups and to evaluate associations with individual Banff lesion scores. The 95% confidence intervals (CIs) for gDSA positivity rates were calculated using the Wilson score interval method, except for groups with complete or absent positivity (100% or 0%), for which the Clopper–Pearson exact method was applied. Odds ratios (ORs) for categorical comparisons were reported with 95% CIs derived from 2 × 2 contingency tables. When any cell contained a zero value, the Haldane–Anscombe continuity correction was applied by adding 0.5 to each cell.

The Mann–Whitney U test was used to compare sDSA and gDSA strength between groups. For continuous quantitative analyses, values below the positivity threshold were imputed as half of the corresponding cutoff (MFI 50 for gDSA), a commonly used approach for handling left-censored data, to reduce distortion from zero inflation and facilitate comparison across recipients (19). Statistical significance was defined as *P <* 0.05 after Bonferroni correction for six pairwise comparisons versus the no-rejection group. In the analysis of the association between histopathologic findings and gDSA, correction for multiple comparisons was not applied because of the exploratory nature of the analysis, and an unadjusted *P* value <0.05 was considered significant.

Continuous variables are presented as median (interquartile range [IQR]), and categorical variables as number (%). Univariable logistic regression analysis was performed to identify factors associated with gDSA positivity. Firth penalized logistic regression was applied in exploratory subgroup analyses with complete separation. A multivariable logistic regression model was subsequently constructed including covariates with a *P* value <0.4 in the univariable analysis. Spearman correlation analysis was performed to evaluate the correlation between the sum of sDSA MFI and the sum of gDSA MFI. All statistical analyses and figure generation were conducted using Python 3.10 (Python Software Foundation, Wilmington, DE, USA).

## 3 Results

### 3.1 Characteristics of the kidney recipients and biopsies

The clinical characteristics of the 60 recipients at the time of transplantation and the 60 biopsies at the time of biopsy (one biopsy per recipient) are summarized in **Table 1**. The median age of recipients at kidney transplantation was 51.5 (42.8–60.0) years. 31 recipients (51.7%) had preformed sDSA prior to transplantation and 23 recipients (38.3%) had desensitization therapy due to preformed sDSA. The median time interval between kidney transplantation and biopsy was 12.4 (0.9–39.8) months. 27 biopsies (45.0%) were performed per protocol while 33 biopsies (55.0%) were performed for cause. The median (IQR) serum DSA strengths for HLA-A, B, DRB1, DR51/52/53, and DQ at the time of biopsy were 2140 (910–2620), 1450 (1312–1629), 1051 (866–2235), 2545 (1955–3432), and 3100 (1431–5264), respectively. The median (IQR) serum DSA strengths for each HLA locus were not significantly different according to histologic findings (**Supplementary Table 1**). However, the frequency of anti-rejection therapy before biopsy was higher in the caABMR+IFTA II group (5/9, 55.6%) than in the other groups (*P* < 0.001), reflecting treatment for previous episodes of ABMR or TCMR. HLA classes of serum and graft DSA are summarized in **Supplementary Table 2**. At the time of biopsy, 11 biopsies (18.3%) showed anti-HLA class I sDSA and 53 biopsies (88.3%) showed anti-HLA class II sDSA.

**Table 1:** Clinical characteristics of 60 recipients and 60 biopsies.

Characteristic	N or median (IQR)
Recipient (N = 60)	
Age, years	51.5 (42.8–60.0)
Female, n (%)	35 (58.3%)
Deceased donor, n (%)	28 (46.7%)
Preformed sDSA, n (%)	31 (51.7%)
Pre-operative desensitization, n (%)	23 (38.3%)
Rituximab	22 (36.7%)
Plasmapheresis	23 (38.3%)
Intravenous immunoglobulin (IVIG)	21 (35.0%)
Re-transplant, n (%)	2 (3.3%)
Induction therapy, n (%)	
Basiliximab	39 (65.0%)
Antithymocyte globulin	21 (35.0%)
None	4 (6.7%)
Maintenance immunosuppression at biopsy, n (%)	
TAC + MMF + CS	42 (70.0%)
TAC + Mizoribine + CS	7 (11.7%)
TAC + mTOR inhibitor	2 (3.3%)
TAC + CS	6 (10.0%)
TAC + MMF + CS + mTOR inhibitor	1 (1.7%)
CsA + MMF + CS	1 (1.7%)
CsA + Mizoribine + CS	1 (1.7%)
Pre-biopsy anti-rejection treatment, n (%)	7 (11.7%)
Biopsies (N = 60)	
TPL–biopsy interval, months	12.4 (0.9–39.8)
For-cause biopsy, n (%)	33 (55.0%)
Biopsy length, cm	0.80 (0.60–1.20)
sDSA specificity, n (%)	
A	7 (11.7%)
B	4 (6.7%)
DRB1	15 (25.0%)
DR51/52/53	12 (20.0%)
DQ	33 (55.0%)
sDSA strength^*^, median MFI (IQR)	
A	2140 (910–2620)
B	1450 (1312–1629)
DRB1	1051 (866–2235)
DR51/52/53	2545 (1955–3432)
DQ	3100 (1431–5264)
sDSA C1q binding positivity^†^, n (%)	2/23 (8.7%)
Histologic diagnosis	
No rejection	22
aABMR	11
aTCMR	7
aABMR+TCMR	6
C4d-only	3
caABMR+IFTA II	9
IFTA III	2

^*^ Among biopsies with detectable sDSA at each locus.

**^†^**sDSA C1q-binding assay results were available in only 23 patients and were selectively ordered at physician discretion, limiting robust analysis.

TAC, tacrolimus; MMF, mycophenolate mofetil; CS, corticosteroids; CsA, cyclosporin.

Desensitization and induction agents were counted independently and were not mutually exclusive; most of the 23 desensitized recipients (21 of 23) received rituximab, therapeutic plasma exchange, and intravenous immunoglobulin in combination, and 4 recipients received both basiliximab and anti-thymocyte globulin as induction.

### 3.2 Presence of gDSA and distribution of HLA loci of DSA according to the graft histologic findings

gDSA positivity according to histologic findings of the 60 graft biopsies is presented in **Table 2**. The gDSA positivity rate differed significantly across histologic groups (*P =* 3.3 × 10^-4^). C4d-only cases showed the highest gDSA positivity rate (100.0%), followed by aABMR (90.9%), aABMR+TCMR (83.3%), and caABMR (44.4%). In contrast, no rejection (22.7%), aTCMR (14.3%) and IFTA III (0.0%) showed lower positive rates, although all 60 cases had positive serum DSA. The gDSA positive rate in the aABMR group was significantly higher than that in the no-rejection group after Bonferroni correction (*Pc =* 0.003; OR 34.0, 95% CI 3.5–334.0). When sensitivity analysis was performed with alternative MFI cutoffs (≥50, ≥150), the gDSA positivity across histologic groups showed comparable results with MFI cutoff ≥100 (**Supplementary Table 3**). The gDSA positive rate in the aABMR group was significantly higher than that in the no-rejection group after Bonferroni correction also with MFI cutoff ≥50 (*Pc* = 0.008) and ≥150 (*Pc =* 0.005).

**Table 2:** gDSA presence by histologic findings (N = 60).

Histologic finding	gDSA+/n	Positive rate (95% CI)	OR (95% CI) vs NR	Corrected P value
No rejection	5/22	22.7% (10.1–43.4%)	(ref)	(ref)
aABMR	10/11	90.9% (62.3–98.4%)	34.0 (3.5–334.0)	4.5e-04 (0.003)
aTCMR	1/7	14.3% (2.6–51.3%)	0.6 (0.1–5.9)	1.000 (1.000)
aABMR+TCMR	5/6	83.3% (43.6–97.0%)	17.0 (1.6–181.0)	0.013 (0.076)
C4d-only	3/3	100.0% (29.2–100.0%)	22.0 (1.0–502.0)	0.024 (0.146)
caABMR+IFTA II	4/9	44.4% (18.9–73.3%)	2.7 (0.5–14.2)	0.385 (1.000)
IFTA III	0/2	0.0% (0.0–84.2%)	0.6 (0.03–15.4)	1.000 (1.000)

All 60 biopsies were sDSA-positive at biopsy. Each histologic group was compared with the no-rejection (NR) group (reference, “ref”) by Fisher’s exact test. 95% CIs were calculated by the Wilson score method (Clopper–Pearson for groups at 0% or 100%). Odds ratios were derived from 2 × 2 tables, with the Haldane–Anscombe correction (+0.5 per cell) applied for zero cells. Corrected *P* values are shown for the six comparisons versus the NR group.

gDSA, intragraft donor-specific antibody; NR, no rejection; aABMR, active antibody-mediated rejection; aTCMR, acute T cell-mediated rejection; caABMR, chronic active ABMR; IFTA, interstitial fibrosis and tubular atrophy; OR, odds ratio; CI, confidence interval; ref, reference group.

In a subset of graft eluates, serologic non-DSA anti-HLA antibodies were observed (**Supplementary Table 4**). However, epitope analysis showed that all serologic non-DSA anti-HLA antibodies shared epitopes with donor HLA antigens and could therefore be considered anti-donor HLA epitope-specific antibodies (DESA). For example, in biopsy #8 of the no rejection group, anti-DQ8 and anti-DQ9 antibody (non-DSA serologically) was detected in biopsy eluate in addition to anti-DQ7 antibody (DSA serologically). These two antibodies were found to target the 55PP epitope commonly present in DQ7, DQ8 and DQ9 antigens. In our cohort, all cases of serologic non-DSA among gDSA were observed only in the presence of concomitant serologic DSA. In 8 biopsies, weak DSAs undetectable in serum at the time of biopsy were identified in graft eluates. In 5 of these cases, the corresponding gDSAs were detected in serum samples obtained either prior to (n=3) or following (n=2) the biopsy (**Supplementary Table 4**).

### 3.3 Factors associated with gDSA positivity

Logistic regression analysis was performed to identify factors associated with the presence of gDSA (**Supplementary Table 5**). In univariable analysis, both the sum of sDSA MFI and the highest sDSA MFI showed trends toward association with gDSA positivity, although neither reached statistical significance (*P* = 0.061 and *P* = 0.069, respectively). In the multivariable logistic regression model including the sum of sDSA, TPL-to-biopsy interval, and age as covariates selected based on a *P* value <0.4 in univariable analysis (the highest sDSA MFI was excluded because of multicollinearity with the sum of sDSA), the sum of sDSA MFI continued to show a trend toward association with gDSA positivity, although the association did not reach statistical significance (*P* = 0.070). Neither the TPL-to-biopsy interval nor age showed a significant association with gDSA positivity. Spearman correlation analysis showed only a weak, non-significant relationship between sum of sDSA and sum of gDSA MFI across the 60 biopsies (rho = 0.205, *P =* 0.116), with no correlation observed within the gDSA-positive subset (rho = 0.059, *P =* 0.77, n = 28).

### 3.4 Strength of graft DSA according to histologic findings

**Figure 1:**
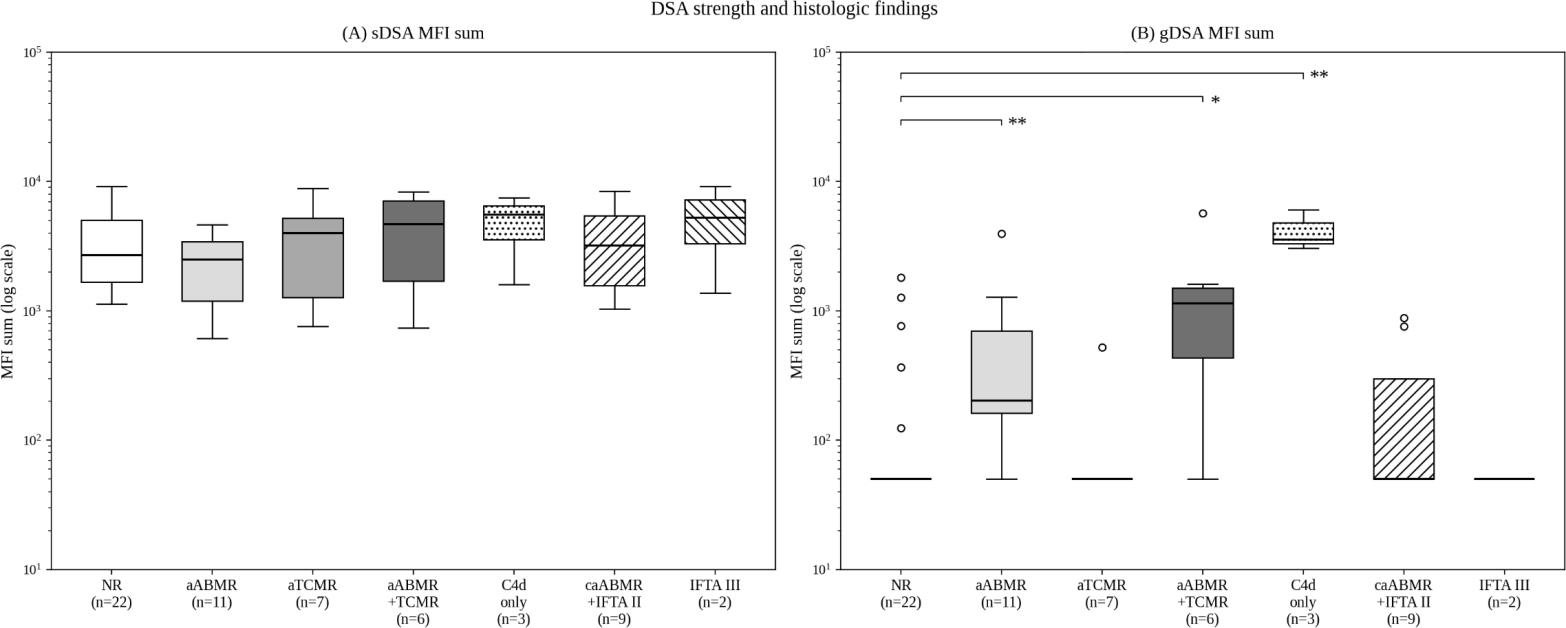
Association between DSA strength and histologic findings in 60 graft biopsies, shown as the MFI sum of **(A)** serum DSA (sDSA) and **(B)** graft DSA (gDSA). For gDSA, values below the positivity threshold were imputed as MFI 50, corresponding to one-half of the biopsy-eluate positivity cutoff of 100. Box plots display median and interquartile range with whiskers extending to 1.5 × IQR; outliers are shown as open circles. *P < 0.05; **P < 0.01 by Mann–Whitney U test with Bonferroni correction for six pairwise comparisons versus the no-rejection group.

The association of gDSA strength with histologic findings is shown in **Figure 1**, where DSA strength is presented as the sum of MFI values. The MFI sum of sDSA was not significantly different according to histologic findings (**Figure 1A**; consistent findings were observed for the highest MFI of sDSA, **Supplementary Figure 1A**). However, the MFI sum of gDSA was significantly different according to histologic lesions. The MFI sum of gDSA in the aABMR group was higher than that in the no-rejection group after Bonferroni correction [median (IQR), 203 (162–700) versus 50 (50–50), *Pc =* 0.009] (**Figure 1B**). Recipients in aABMR+TCMR and C4d-only groups also showed higher gDSA MFI sums compared to no-rejection group after correction [1140 (432–1509) versus 50 (50–50), *Pc =* 0.035; 3569 (3318–4815) versus 50 (50–50), *Pc =* 0.007, respectively]. Otherwise, aTCMR, caABMR, and IFTA III groups did not differ significantly from no-rejection group in the sum of gDSA MFI. When analyses were performed using the highest MFI value of gDSA, consistent results were observed (**Supplementary Figure 1B**). The highest MFI of gDSA in the aABMR, aABMR+TCMR and C4d-only groups were higher than that in the no-rejection group after Bonferroni correction (*Pc =* 0.004, *Pc =* 0.028 and *Pc =* 0.007, respectively). In addition, to account for variability in biopsy tissue amount, the gDSA MFI sum was normalized to the cohort mean biopsy length. After normalization, the adjusted gDSA MFI sum also showed consistent results, remained higher in the aABMR, aABMR+TCMR, and C4d-only groups than in the no-rejection group after Bonferroni correction (*Pc =* 0.009, *Pc =* 0.019, and *Pc =* 0.007, respectively; **Supplementary Figure 2A**). When using MFI sum of graft donor HLA epitope-specific antibodies (DESA), MFI sum of graft DESA was higher only in aABMR group than in no-rejection group after Bonferroni correction (*Pc* = 0.034), with marginal significance in aABMR+TCMR group (*Pc* = 0.058) (**Supplementary Figure 2B**).

### 3.5 Association between gDSA and Banff lesion scores

**Figure 2:**
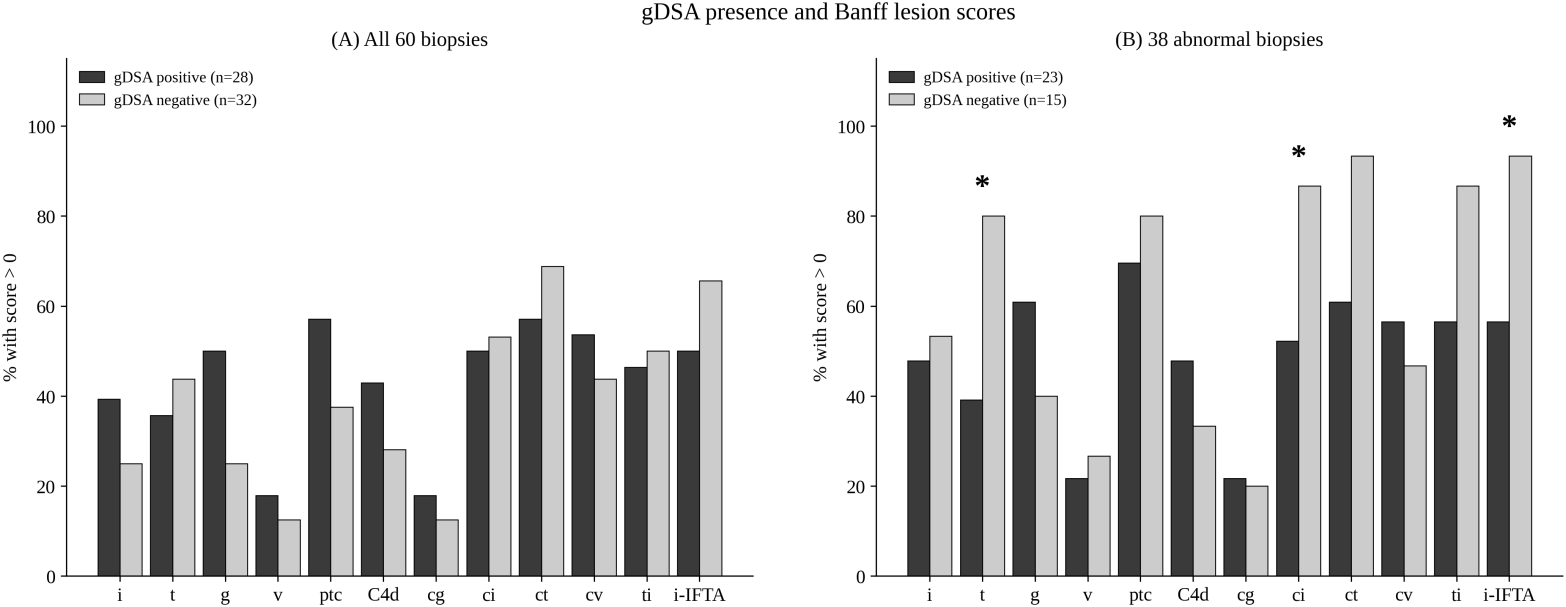
Association between the presence of gDSA and Banff lesion scores in **(A)** all 60 graft biopsies and **(B)** the 38 abnormal graft biopsies (excluding the no-rejection group). Bars show the proportion of biopsies with a Banff lesion score ≥ 1, comparing gDSA-positive and gDSA-negative biopsies (open bars, gDSA-negative; hatched gray bars, gDSA-positive). *P < 0.05 by Fisher’s exact test. No Bonferroni correction was applied as this was an exploratory analysis.

The distribution of gDSA according to each Banff lesion score is depicted in **Figure 2**. The presence of gDSA was not associated with any Banff lesion score in the 60 biopsies (**Figure 2A**). Because gDSA positivity in chronic lesions resembled that of the no-rejection group, we restricted the analysis to the 38 abnormal biopsies to better delineate lesion-specific associations (**Figure 2B**). In the 38 abnormal biopsies, gDSA-positive biopsies showed a significantly lower positivity of t score (≥1) (tubulitis) compared with gDSA-negative biopsies (39.1% versus 80.0%, *P =* 0.020), as well as ci (52.2% versus 86.7%, *P =* 0.039), and i-IFTA (56.5% versus 93.3%, *P =* 0.026). These findings should be interpreted with caution as no correction for multiple comparisons was applied in this exploratory analysis.

## 4 Discussion

Although circulating sDSA has been widely used as a surrogate marker of alloimmune risk (20), in this study the strength of sDSA showed only a marginal association with gDSA positivity, and the correlation between the strength of sDSA and gDSA was weak. These findings reinforce the concept that intragraft antibody binding is not merely a quantitative reflection of circulating antibody burden. Moreover, sDSA alone may not adequately reflect actual antibody engagement within the graft microvasculature or the accompanying histopathologic manifestations of injury. The C1q-binding capacity of sDSA was also not significantly associated with gDSA positivity or specific histologic lesions, although this analysis was limited by the small and selectively tested subset (n = 23).

In this study of kidney transplant recipients with concurrent serum DSA, we observed that intragraft donor-specific antibodies were differentially distributed across histologic phenotypes. Our findings suggest that gDSA more closely reflects antibody–graft interaction within the tissue microenvironment than sDSA alone (21, 22). Notably, gDSA was most frequently and strongly detected in biopsies with aABMR-related lesions, whereas its detection was limited in both no-rejection and chronic lesion groups. Our results are partially consistent with prior studies, although some differences should be noted. Bachelet et al. demonstrated associations between gDSA and AMR-related microcirculatory lesions, including ptc, g, and cg scores (15). Similarly, Nocera et al. reported associations between gDSA and AMR-related histologic features, although these relationships were attenuated when analysis was restricted to recipients with positive sDSA (16). In contrast, Courant et al. did not observe a clear association between gDSA and antibody-mediated lesions (17). These discrepancies may be attributable to differences in study design and patient selection, as well as the relatively small sample sizes. Notably, our study specifically included only recipients with concurrent sDSA, enabling a more focused evaluation of how gDSA relates to histologic heterogeneity within this clinically relevant population.

Conversely, the prevalence of gDSA in the aTCMR group was low, consistent with previous reports (15). Previous studies have suggested that TCMR may be associated with the development of AMR through complex immune reactions, including T-cell activation (23, 24). However, in this small aTCMR subgroup, circulating DSA was infrequently detected as graft-bound DSA at the time of biopsy. This may be because, in TCMR, where the immune response is primarily T cell–mediated and injury is largely confined to the tubulointerstitial compartment, endothelial activation and HLA expression in the graft microvasculature are relatively limited, potentially restricting antibody binding.

Similarly, gDSA detection was not enriched in caABMR with IFTA II or advanced IFTA in this cohort, in contrast to its higher frequency in active ABMR-related lesions. This pattern should be interpreted in the context of differences in post-transplant timing and chronic tissue remodeling across histologic groups. Recent transcriptomic data suggest that caABMR may share molecular features with TCMR rather than simply representing a late counterpart of aABMR (25). In addition, caABMR in the present cohort was mostly accompanied by IFTA grade II, and advanced IFTA showed no detectable gDSA in this small cohort. A recent single-cell transcriptomic study demonstrated that IFTA showed an increased gene expression of extracellular matrix formation but decreased population of macrophages characterized by upregulated HLA class II genes, findings that may suggest a less active immunological state in fibrotic grafts (26). These observations suggest that the immunologic milieu required for effective antibody binding may be less readily sustained in chronically remodeled grafts, though this interpretation remains speculative in the absence of longitudinal or functional data.

Notably, additional insight into these processes is provided by the behavior of HLA-DQ–specific antibodies. In the no rejection group, DQ-specific DSAs were frequently detected in serum (15 of 22 cases), whereas only 3 cases were identified as gDSA. HLA-DQ expression on renal microvasculature endothelium has been reported to be lower than that of HLA-DR under baseline conditions but inducible under inflammatory stimuli such as interferon-gamma (27, 28). These findings raise the possibility that, in the no-rejection group, relatively preserved graft tissue may be associated with limited antigen availability or reduced endothelial activation, which could restrict antibody binding; however, this remains speculative and cannot be inferred causally from the present cross-sectional dataset.

Moreover, in a subset of biopsies, weak DSAs that were not detectable in serum at the time of biopsy were identified in graft eluates. Some of these antibodies had been observed in serum at earlier time points, suggesting that gDSA may capture locally retained antibodies or reflect prior antibody binding retained within the graft despite being undetectable in contemporaneous serum measurements. Further studies using longitudinal samples may provide additional insight into local antibody dynamics within the graft.

Additionally, epitope analysis of graft eluates showed that all non-DSA anti-HLA antibodies shared epitopes with donor HLA antigens, confirming the presence of donor HLA epitope-specific antibodies at the tissue level. However, these donor HLA epitope-specific antibodies were observed only in the presence of conventional serologic gDSA in this study, suggesting that serologic gDSA analysis may still retain practical relevance in the assessment of donor-specific humoral immunity.

In contrast to some previous studies, we did not observe a significant association between gDSA and microvascular inflammation scores such as glomerulitis or peritubular capillaritis. This discrepancy may be partly explained by the inclusion of C4d-only cases, which exhibited high gDSA positivity without corresponding increases in these lesion scores, although this subgroup was very small (n = 3). These findings suggest that antibody binding may occur independently of overt histologic injury, and additional factors may be required for the development of overt antibody-mediated injury (29, 30). In the abnormal-biopsy subset, lower frequencies of tubulitis and chronic tubulointerstitial injury scores (ci and i-IFTA) were observed among gDSA-positive biopsies, consistent with the lower gDSA positivity observed in the TCMR and caABMR+IFTA II groups. These findings further support the interpretation that gDSA detection may preferentially reflect antibody-mediated tissue interaction rather than TCMR- or fibrosis-dominant injury patterns, although these findings should be interpreted cautiously because no correction for multiple comparisons was applied.

This study has several limitations. First, the sample size was relatively small, particularly within individual histologic subgroups, limiting statistical power and precluding definitive subgroup-level conclusions. Second, only recipients with detectable sDSA and available frozen biopsy tissue were included. Third, variability in biopsy tissue size may have influenced gDSA quantification, although normalization yielded consistent results. Fourth, anti-rejection therapy administered prior to biopsy, particularly in the caABMR+IFTA II group, may have influenced both circulating sDSA and intragraft gDSA levels, potentially confounding their interpretation. Fifth, because gDSA is obtained from biopsy tissue, it is not intended to serve as a substitute for histopathologic diagnosis, but rather as a complementary tool for immunologic assessment. Although the additional reagent cost for gDSA analysis may not be substantial, the requirement for frozen biopsy tissue, additional personnel resources, and the lack of established technical standardization across centers and prospective clinical validation data may limit its implementation in routine clinical practice. Future prospective studies with standardized protocols are needed to evaluate its clinical utility.

Nevertheless, unlike previous studies, our cohort included a broad spectrum of post-transplant histologic phenotypes, including caABMR and advanced IFTA, enabling evaluation of gDSA across diverse patterns of allograft injury. Our findings suggest that intragraft antibody binding is not solely explained by the presence, strength, or complement binding activity of circulating DSA, but may be associated with differences in the local graft environment. These findings contribute to a more comprehensive understanding of antibody–graft interactions in the post-transplant setting. Further studies with larger cohorts and longitudinal designs are needed to better define the role of gDSA in the pathogenesis of kidney allograft injury and to elucidate the mechanisms that determine when circulating antibodies engage the graft and how such interactions translate into tissue injury.

## Data Availability

The original contributions presented in the study are included in the article/**Supplementary Material**. Further inquiries can be directed to the corresponding authors.
