# Supplementary material for: Intragraft donor-specific antibodies reflect histologic heterogeneity in kidney allografts with concurrent serum DSA
Source: Front Immunol. 2026 Jun 23;17:1878975. doi: 10.3389/fimmu.2026.1878975 (PMC13337509; doi:10.3389/fimmu.2026.1878975)

# SUPPLEMENTARY TABLES

## Supplementary Table 1. Clinical characteristics of 60 recipients and 60 biopsies according to the graft histologic findings

| Characteristic | No rejection | aABMR | aTCMR | aABMR+TCMR | C4d-only | caABMR+IFTA II | IFTA III | *P* value |
| --- | --- | --- | --- | --- | --- | --- | --- | --- |
| Recipients (N=60) |  |  |  |  |  |  |  |  |
| Age, years | 56.5 (47.2–61.0) | 57.0 (48.0–60.0) | 55.0 (40.5–61.5) | 32.5 (24.5–45.0) | 50.0 (49.5–50.5) | 45.0 (43.0–52.0) | 47.0 (46.0–48.0) | 0.176 |
| Female, n (%) | 12 (54.5%) | 6 (54.5%) | 3 (42.9%) | 5 (83.3%) | 3 (100.0%) | 5 (55.6%) | 1 (50.0%) | 0.589 |
| Deceased donor, n (%) | 15 (68.2%) | 4 (36.4%) | 2 (28.6%) | 3 (50.0%) | 2 (66.7%) | 2 (22.2%) | 0 (0.0%) | 0.129 |
| Preformed sDSA, n (%) | 12 (54.5%) | 6 (54.5%) | 2 (28.6%) | 5 (83.3%) | 3 (100.0%) | 3 (33.3%) | 0 (0.0%) | 0.118 |
| Re-transplant, n (%) | 1 (4.5%) | 0 (0.0%) | 0 (0.0%) | 1 (16.7%) | 0 (0.0%) | 0 (0.0%) | 0 (0.0%) | 0.607 |
| Desensitization, n (%) | 10 (45.5%) | 5 (45.5%) | 1 (14.3%) | 1 (16.7%) | 3 (100.0%) | 3 (33.3%) | 0 (0.0%) | 0.134 |
| RTX | 10 (45.5%) | 5 (45.5%) | 1 (14.3%) | 0 (0.0%) | 3 (100.0%) | 3 (33.3%) | 0 (0.0%) | 0.052 |
| TPE | 10 (45.5%) | 5 (45.5%) | 1 (14.3%) | 1 (16.7%) | 3 (100.0%) | 3 (33.3%) | 0 (0.0%) | 0.134 |
| IVIG | 10 (45.5%) | 5 (45.5%) | 1 (14.3%) | 0 (0.0%) | 2 (66.7%) | 3 (33.3%) | 0 (0.0%) | 0.201 |
| Induction, n (%) |  |  |  |  |  |  |  |  |
| Basiliximab | 14 (63.6%) | 5 (45.5%) | 6 (85.7%) | 6 (100.0%) | 1 (33.3%) | 5 (55.6%) | 2 (100.0%) | 0.164 |
| ATG | 9 (40.9%) | 6 (54.5%) | 1 (14.3%) | 1 (16.7%) | 3 (100.0%) | 1 (11.1%) | 0 (0.0%) | 0.039 |
| None | 0 (0.0%) | 0 (0.0%) | 1 (14.3%) | 0 (0.0%) | 0 (0.0%) | 3 (33.3%) | 0 (0.0%) | 0.029 |
| Maintenance at biopsy, n (%) |  |  |  |  |  |  |  |  |
| TAC + MMF + CS | 17 (77.3%) | 6 (54.5%) | 6 (85.7%) | 3 (50.0%) | 3 (100.0%) | 7 (77.8%) | 0 (0.0%) | 0.092 |
| TAC + Mizoribine + CS | 1 (4.5%) | 3 (27.3%) | 1 (14.3%) | 0 (0.0%) | 0 (0.0%) | 1 (11.1%) | 1 (50.0%) | 0.198 |
| TAC + mTOR inhibitor | 0 (0.0%) | 1 (9.1%) | 0 (0.0%) | 1 (16.7%) | 0 (0.0%) | 0 (0.0%) | 0 (0.0%) | 0.247 |
| TAC + CS | 3 (13.6%) | 1 (9.1%) | 0 (0.0%) | 1 (16.7%) | 0 (0.0%) | 0 (0.0%) | 1 (50.0%) | 0.296 |
| TAC + MMF + CS + mTOR inhibitor | 1 (4.5%) | 0 (0.0%) | 0 (0.0%) | 0 (0.0%) | 0 (0.0%) | 0 (0.0%) | 0 (0.0%) | 0.459 |
| CsA + MMF + CS | 0 (0.0%) | 0 (0.0%) | 0 (0.0%) | 0 (0.0%) | 0 (0.0%) | 1 (11.1%) | 0 (0.0%) | 0.190 |
| CsA + Mizoribine + CS | 0 (0.0%) | 0 (0.0%) | 0 (0.0%) | 1 (16.7%) | 0 (0.0%) | 0 (0.0%) | 0 (0.0%) | 0.089 |
| Anti-rejection treatment before biopsy, n (%) | 0 (0.0%) | 0 (0.0%) | 0 (0.0%) | 1 (16.7%) | 0 (0.0%) | 5 (55.6%) | 1 (50.0%) | <0.001 |
| Interval from anti-rejection therapy to biopsy (median, range) (months) | NA | NA | NA | 3 (n=1) | NA | 33 (7~72) | 105 (n=1) |  |
| Steroid pulse | 0 (0.0%) | 0 (0.0%) | 0 (0.0%) | 1 (16.7%) | 0 (0.0%) | 4 (44.4%) | 1 (50.0%) | 0.002 |
| TPE | 0 (0.0%) | 0 (0.0%) | 0 (0.0%) | 0 (0.0%) | 0 (0.0%) | 2 (22.2%) | 0 (0.0%) | 0.068 |
| IVIG | 0 (0.0%) | 0 (0.0%) | 0 (0.0%) | 0 (0.0%) | 0 (0.0%) | 1 (11.1%) | 0 (0.0%) | 0.450 |
| RTX | 0 (0.0%) | 0 (0.0%) | 0 (0.0%) | 0 (0.0%) | 0 (0.0%) | 3 (33.3%) | 0 (0.0%) | 0.007 |
| ATG | 0 (0.0%) | 0 (0.0%) | 0 (0.0%) | 1 (16.7%) | 0 (0.0%) | 0 (0.0%) | 0 (0.0%) | 0.165 |
| Biopsies (N=60) |  |  |  |  |  |  |  |  |
| TPL–Bx interval, months | 8.1 (0.7–14.5) | 12.7 (10.3–21.7) | 12.9 (2.1–25.6) | 11.8 (0.3–33.3) | 0.3 (0.3–0.3) | 64.6 (13.2–163.5) | 109.9 (90.6–129.2) | 0.015 |
| For-cause biopsy, n (%) | 10 (45.5%) | 7 (63.6%) | 4 (57.1%) | 3 (50.0%) | 0 (0.0%) | 7 (77.8%) | 2 (100.0%) | 0.210 |
| Biopsy length, cm | 0.9 (0.6–1.2) | 0.7 (0.5–0.9) | 0.8 (0.7–1.1) | 0.9 (0.8–1.1) | 0.6 (0.6–0.9) | 0.8 (0.7–1.3) | 0.9 (0.8–1.1) | 0.726 |
| Serum creatinine (mg/dL) | 1.1 (0.9–1.4) | 1.2 (0.8–1.4) | 1.1 (1.0–1.3) | 3.2 (1.7–4.8) | 0.6 (0.6–0.7) | 2.1 (2.0–2.2) | 2.6 (2.6–2.6) | 0.028 |
| eGFR (mL/min/1.73 m²) | 60.8 (48.6–92.6) | 63.8 (48.9–85.1) | 61.7 (57.0–62.9) | 20.3 (12.2–40.4) | 103.5 (100.4–106.7) | 35.1 (30.2–40.0) | 21.2 (21.2–21.2) | 0.024 |
| sDSA strength (A) | 2140 (1638–2559) | 1437 (1025–1849) | — | — | — | 4019 (2352–5686) | — | 0.738 |
| sDSA strength (B) | 1221 (1221–1221) | 1557 (1450–1700) | — | — | — | — | — | — |
| sDSA strength (DRB1) | 1051 (942–1574) | 972 (837–1376) | 757 (757–757) | 1242 (990–4802) | 5184 (5010–5357) | 1290 (1151–1428) | — | 0.411 |
| sDSA strength (DR51/52/53) | 3262 (2333–4754) | 2657 (2657–2657) | 3649 (2390–4908) | — | 2118 (1861–2374) | 2459 (1746–2834) | — | 0.796 |
| sDSA strength (DQ) | 2406 (1522–5160) | 3063 (1028–3099) | 4128 (3346–5422) | 6229 (4664–6812) | — | 3157 (1208–5105) | 5262 (3319–7204) | 0.558 |
| sDSA C1q binding positivity^*^, n (%) | 1/11 (9.1%) | 0/2 (0.0%) | 1/3 (33.3%) | 0/2 (0.0%) | 0/2 (0.0%) | 0/2 (0.0%) | 0/1 (0.0%) | 0.789 |

Data are presented as median (interquartile range) or number of recipients or biopsies (percentages). Overall *P* values are from Kruskal–Wallis test for continuous variables and chi-square test for categorical variables. ^*^sDSA C1q-binding assay results were available in only 23 patients and were selectively ordered at physician discretion, limiting robust analysis.

Abbreviations: aABMR, active antibody-mediated rejection; aTCMR, acute T-cell mediated rejection; caABMR, chronic active antibody-mediated rejection; IFTA, interstitial fibrosis and tubular atrophy; HLA, human leukocyte antigen; sDSA, serum donor-specific antibodies. RTX, rituximab; TPE, therapeutic plasma exchange; IVIG, intravenous immunoglobulin; ATG, anti-thymocyte globulin; TAC, tacrolimus; MMF, mycophenolate mofetil; CS, corticosteroids; CsA, cyclosporin; NA, not applicable.

Desensitization, induction, and anti-rejection treatment modalities were counted independently and were not mutually exclusive; most of the 23 desensitized recipients (21 of 23) received rituximab, therapeutic plasma exchange, and intravenous immunoglobulin in combination, and 4 recipients received both basiliximab and anti-thymocyte globulin as induction.

## Supplementary Table 2. Distribution of HLA classes of DSA according to the histologic findings in the 60 graft biopsies

| **HLA classes of DSA** | **No rejection**  **(n = 22)** | **aABMR**  **(n = 11)** | **aTCMR**  **(n = 7)** | **aABMR+TCMR**  **(n = 6)** | **C4d-only**  **(n = 3)** | **caABMR+IFTA II**  **(n = 9)** | **IFTA III**  **(n = 2)** |
| --- | --- | --- | --- | --- | --- | --- | --- |
| sDSA at biopsy |  |  |  |  |  |  |  |
| A | 3 (13.6%) | 2 (18.2%) | 0 (0.0%) | 0 (0.0%) | 0 (0.0%) | 2 (22.2%) | 0 (0.0%) |
| B | 1 (4.5%) | 3 (27.3%) | 0 (0.0%) | 0 (0.0%) | 0 (0.0%) | 0 (0.0%) | 0 (0.0%) |
| DRB1 | 3 (13.6%) | 4 (36.4%) | 1 (14.3%) | 3 (50.0%) | 2 (66.7%) | 2 (22.2%) | 0 (0.0%) |
| DR51/52/53 | 4 (18.2%) | 1 (9.1%) | 2 (28.6%) | 0 (0.0%) | 2 (66.7%) | 3 (33.3%) | 0 (0.0%) |
| DQ | 15 (68.2%) | 5 (45.5%) | 4 (57.1%) | 3 (50.0%) | 0 (0.0%) | 4 (44.4%) | 2 (100.0%) |
| gDSA at biopsy |  |  |  |  |  |  |  |
| A | 0 (0.0%) | 1 (9.1%) | 0 (0.0%) | 0 (0.0%) | 0 (0.0%) | 1 (11.1%) | 0 (0.0%) |
| B | 0 (0.0%) | 3 (27.3%) | 0 (0.0%) | 0 (0.0%) | 0 (0.0%) | 0 (0.0%) | 0 (0.0%) |
| DRB1 | 0 (0.0%) | 3 (27.3%) | 0 (0.0%) | 3 (50.0%) | 3 (100.0%) | 1 (11.1%) | 0 (0.0%) |
| DR51/52/53 | 2 (9.1%) | 1 (9.1%) | 0 (0.0%) | 0 (0.0%) | 0 (0.0%) | 0 (0.0%) | 0 (0.0%) |
| DQ | 3 (13.6%) | 2 (18.2%) | 1 (14.3%) | 2 (33.3%) | 0 (0.0%) | 2 (22.2%) | 0 (0.0%) |

Data are presented as number of biopsies (percentages of group total).

Abbreviations: sDSA, serum donor-specific antibodies; gDSA, intragraft donor-specific antibodies; aABMR, active antibody-mediated rejection; aTCMR, acute T cell-mediated rejection; caABMR, chronic active ABMR; IFTA, interstitial fibrosis and tubular atrophy.

## Supplementary Table 3. Sensitivity analysis of gDSA positivity by histologic findings at alternative MFI cutoffs.

| **Histologic finding** | **MFI ≥50** | **MFI ≥ 100 (primary)** | **MFI ≥ 150** |
| --- | --- | --- | --- |
| No rejection (n = 22) | 7/22 (31.8%) | 5/22 (22.7%) | 4/22 (18.2%) |
| aABMR (n = 11) | 10/11 (90.9%) | 10/11 (90.9%) | 9/11 (81.8%) |
| aTCMR (n = 7) | 1/7 (14.3%) | 1/7 (14.3%) | 1/7 (14.3%) |
| aABMR+TCMR (n = 6) | 6/6 (100.0%) | 5/6 (83.3%) | 5/6 (83.3%) |
| C4d-only (n = 3) | 3/3 (100.0%) | 3/3 (100.0%) | 3/3 (100.0%) |
| caABMR+IFTA II (n = 9) | 5/9 (55.6%) | 4/9 (44.4%) | 4/9 (44.4%) |
| IFTA III (n = 2) | 0/2 (0.0%) | 0/2 (0.0%) | 0/2 (0.0%) |
| Total positivity | 32/60 (53.3%) | 28/60 (46.7%) | 26/60 (43.3%) |
| aABMR vs NR: OR [95% CI]  (corrected *P*) | 21.4 [2.2–204.9]  (*Pc* = 0.008) | 34.0 [3.5–334.0]  (*Pc* = 0.003) | 20.3 [3.1–132.3]  (*Pc* = 0.005) |

OR, odds ratio (aABMR vs no rejection) by Fisher's exact test; NR, no rejection; aABMR, active antibody-mediated rejection; aTCMR, acute T cell-mediated rejection; caABMR, chronic active ABMR; IFTA, interstitial fibrosis and tubular atrophy; MFI, mean fluorescence intensity.

## Supplementary Table 4. HLA specificities of serum and graft DSA and epitope analysis of graft DSA in 60 graft biopsies

| Biopsy group/  number | sDSA at biopsy^*^ | | | gDSA^*^ | | | | gDSA not detected at serum at biopsy  (but observed at other time) | | Graft non-DSA^†^ | | | Epitope analysis of gDSA^‡^ | |  |
| --- | --- | --- | --- | --- | --- | --- | --- | --- | --- | --- | --- | --- | --- | --- | --- |
|  | **Class I** | **Class II** | | **Class I** | | **Class II** | |  |  |  |  |  |  |  |  |
| No rejection (n = 22) | | | | |  | |  | |  | |  | | |  | |
| 1 | Negative | DQ8 (1431) | | Negative | | Negative | | None | | Negative | | | NA | |  |
| 2 | Negative | DQ2 (4840) | | Negative | | Negative | | None | | Negative | | | NA | |  |
| 3 | B60 (1221); *C*15:02* (1312) | DR14 (1051); DR52 (2072) | | Negative | | Negative | | None | | Negative | | | NA | |  |
| 4 | A2 (2978) | Negative | | Negative | | Negative | | None | | Negative | | | NA | |  |
| 5 | Negative | DQ6 (2406) | | Negative | | Negative | | None | | Negative | | | NA | |  |
| 6 | Negative | DQ4 (7650) | | Negative | | DQ4 (1274) | | None | | DQ2, DQ5; DQ6; DQ7 | | | 52PR; 25YT, 74SV2 | |  |
| 7 | Negative | DQ8 (1944) | | Negative | | Negative | | None | | Negative | | | NA | |  |
| 8 | Negative | DR1 (832); DQ7 (7126) | | Negative | | DQ7 (1811) | | None | | DQ8; DQ9 | | | 55PP | |  |
| 9 | Negative | DQ9 (1590) | | Negative | | Negative | | None | | Negative | | | NA | |  |
| 10 | Negative | DQ4 (1474) | | Negative | | Negative | | None | | Negative | | | NA | |  |
| 11 | Negative | DR51 (2420) | | A2 (122); B35 (120) | | Negative | | A2 (never); B35 (never) | | B51 | | | 43Q+62GER, 44RT+69TNT, 97V | |  |
| 12 | Negative | DQ9 (1177) | | Negative | | Negative | | None | | Negative | | | NA | |  |
| 13 | Negative | DR53 (4103) | | Negative | | DR53 (124) | | None | | Negative | | | 48Q6 | |  |
| 14 | Negative | DQ4 (9195) | | Negative | | DR4 (374); DQ4 (329) | | DR4 (never) | | DQ7, DQ8, DQ9 | | | 96Y2, 52PL3 | |  |
| 15 | Negative | DR9 (2097) | | Negative | | Negative | | None | | Negative | | | NA | |  |
| 16 | Negative | DQ4 (3641) | | Negative | | Negative | | None | | Negative | | | NA | |  |
| 17 | Negative | DQ5 (1571) | | Negative | | Negative | | None | | Negative | | | NA | |  |
| 18 | A24 (1136) | Negative | | Negative | | Negative | | None | | Negative | | | NA | |  |
| 19 | A24 (2140) | Negative | | Negative | | Negative | | None | | Negative | | | NA | |  |
| 20 | Negative | DQ9 (5254) | | Negative | | Negative | | None | | Negative | | | NA | |  |
| 21 | Negative | DQ9 (5056) | | Negative | | Negative | | None | | Negative | | | NA | |  |
| 22 | Negative | DR53 (6706) | | Negative | | Negative | | None | | Negative | | | NA | |  |
| aABMR (n = 11) | | | | |  | |  | |  | |  | | |  | |
| 1 | B7 (1844) | DR1 (648) | | A24 (160) | | Negative | | A24 (14 months prior to biopsy) | | A23 | | | 65GK | |  |
| 2 | Negative | DR53 (2657) | | Negative | | DR4 (360); DR53 (254) | | DR4 (8 months prior to biopsy) | | DR1 | | | 96Y2, 70QRA, 48Q6 | |  |
| 3 | Negative | DR12 (1043) | | Negative | | DR12 (164) | | None | | DR8 | | | 96HK, 16Y | |  |
| 4 | A24 (2261) | DR15 (2373) | | A3 (440); A24 (174) | | DR15 (171) | | A3 (3 months post biopsy) | | A23, B37 | | | 65GK, 161D | |  |
| 5 | A1 (613) | Negative | | A1 (135) | | Negative | | None | | Negative | | | 163RG | |  |
| 6 | B51 (1557) | Negative | | B51 (1109) | | Negative | | None | | B13, B18, B35, B38, B49,  B52, B53, B57, B58, B59,  B62, B63, B71, B72, B75,  B77, B78 | | | 163LW+65QIT, 44RT,  80I+69TNT | |  |
| 7 | B51 (1342) | Negative | | B51 (3969) | | Negative | | None | | A32, A23, A24, A25, A30,  A31, B13, B27, B35, B37,  B38, B44, B47, B49, B51,  B52, B53, B57, B58, B59,  B63, B77, B78 | | | 82LR, 44RT+69TNT, 56R | |  |
| 8 | Negative | DR4 (900); DQ4 (3063) | | Negative | | DR4 (169) | | None | | Negative | | | 96Y2 | |  |
| 9 | Negative | DQ6 (1028) | | Negative | | Negative | | None | | Negative | | | Negative | |  |
| 10 | Negative | DQ5 (1691); DQ6 (2066) | | Negative | | DQ5 (165); DQ6 (147) | | None | | Negative | | | 55RPD, 125SQ | |  |
| 11 | Negative | DQ6 (3099) | | Negative | | DQ6 (203) | | None | | Negative | | | Not defined | |  |
| C4d-only (n = 3) | | | | |  | |  | |  | |  | | |  | |
| 1 | Negative | DR8 (5531) | | Negative | | DR8 (3569) | | None | | Negative | | | 74L | |  |
| 2 | Negative | DR10 (4836);  DR51 (2631) | | Negative | | DR10 (5961); DR51 (100) | | None | | DR1, DR9, DR103 | | | 13FE, 40YD2, 96EV | |  |
| 3 | Negative | DR53 (1604) | | Negative | | DR4 (91); DR7 (2976) | | DR4 (1-month prior biopsy)  DR7 (1-month prior biopsy) | | Negative | | | 25Q, 96Y | |  |
| aABMR+TCMR (n = 6) | | | | |  | |  | |  | |  | | |  | |
| 1 | Negative | DQ7 (6229) | | Negative | | DQ7 (1608) | | None | | DQ8, DQ9 | | | 55PP | |  |
| 2 | Negative | DR1 (717); DR10 (7646) | | Negative | | DR1 (170); DR10 (1041) | | None | | DR53 | | | 13FEL; 96QK2 | |  |
| 3 | Negative | DR8 (737) | | Negative | | DR8 (655); DR52 (227);  DQ6 (120) | | DR52 (never);  DQ6 (1-month post biopsy) | | DR11, DR12, DR13, DR14,  DR15, DR16, DR17, DR18,  DQ5 | | | 149H, 13SE, 142M3, 52PQ2 | |  |
| 4 | Negative | DR7 (1242) | | Negative | | DR7 (2959); DQ2 (2725) | | DQ2 (never) | | DQ4, DQ7, DQ8, DQ9 | | | 25Q3; 45GE3, 47KHL | |  |
| 5 | Negative | DQ7 (7395) | | Negative | | DQ7 (220) | | None | | DQ2, DQ8, DQ9 | | | 55PP | |  |
| 6 | Negative | DQ5 (3100) | | Negative | | Negative | | None | | Negative | | | NA | |  |
| aTCMR (n = 7) | | | | |  | |  | |  | |  | | |  | |
| 1 | Negative | DR52 (6166) | | Negative | | Negative | | None | | Negative | | | NA | |  |
| 2 | Negative | DR9 (757) | | Negative | | Negative | | None | | Negative | | | NA | |  |
| 3 | Negative | DQ8 (8903) | | Negative | | DQ8 (521) | | None | | DQ7, DQ9 | | | 55PP | |  |
| 4 | Negative | DR51 (1132) | | Negative | | Negative | | None | | Negative | | | NA | |  |
| 5 | Negative | DQ8 (3994) | | Negative | | Negative | | None | | | Negative | | NA | |  |
| 6 | Negative | DQ2 (1400) | | Negative | | Negative | | None | | | Negative | | NA | |  |
| 7 | Negative | DQ6 (4262) | | Negative | | Negative | | None | | | Negative | | NA | |  |
| caABMR+IFTA II (n = 9) | | | | |  | |  | |  | | |  | |  | |
| 1 | Negative | DR10 (1567) | | Negative | | DR10 (882) | | None | | | Negative | | 40YD2 | |  |
| 2 | Negative | DR51 (2459); DQ6 (864) | | Negative | | Negative | | None | | | Negative | | NA | |  |
| 3 | A31 (7353) | Negative | | A31 (298) | | Negative | | None | | | A31, B48, A33 | | 73ID | |  |
| 4 | Negative | DQ7 (1332) | | Negative | | DQ7 (757) | | None | | | DQ2, DQ4 | | 40GR | |  |
| 5 | Negative | DQ6 (5443) | | Negative | | Negative | | None | | | Negative | | NA | |  |
| 6 | Negative | DQ7 (4992) | | Negative | | DQ7 (183) | | None | | | DQ2, DQ4 | | 40GR | |  |
| 7 | Negative | DR51 (3209) | | Negative | | Negative | | None | | | Negative | | NA | |  |
| 8 | Negative | DR51 (1032) | | Negative | | Negative | | None | | | Negative | | NA | |  |
| 9 | A31 (685) | DR12 (1012) | | Negative | | Negative | | None | | | Negative | | NA | |  |
| IFTA III (n = 2) | | |  | |  | |  | |  | | |  | |  | |
| 1 | Negative | DQ4 (1377) | | Negative | | Negative | | None | | | Negative | | NA | |  |
| 2 | Negative | DQ7 (9146) | | Negative | | Negative | | None | | | Negative | | NA | |  |

^*^The MFI values of each sDSA and gDSA are shown in parentheses. ^†^ Non-DSAs observed in graft eluates shared common epitopes with DSAs and were therefore considered donor HLA epitope-specific antibodies. ^‡^Shared epitopes between gDSAs and graft non-DSAs are shown. Epitope analysis was not applicable when gDSA was negative. "Negative" indicates values below the cutoff thresholds (<500 for sDSA and <100 for gDSA and graft non-DSA).

Abbreviations: HLA, human leukocyte antigens; DSA, donor-specific antibodies; sDSA, serum donor-specific antibodies; gDSA, intragraft donor-specific antibodies; MFI, mean fluorescence intensity; NA, not applicable.

## Supplementary Table 5. Univariable and multivariable logistic regression analyses of factors associated with the presence of intragraft donor-specific antibodies (gDSA) in the 60 graft biopsies.

| **Variable** | **OR (95% CI)** | ***P* value** |
| --- | --- | --- |
| **Univariable analysis (n = 60)** | | |
| Age, per year | 0.98 (0.94–1.02) | 0.360 |
| Female | 1.20 (0.43–3.37) | 0.726 |
| Deceased donor | 0.98 (0.36–2.71) | 0.972 |
| Preformed sDSA | 1.51 (0.54–4.19) | 0.428 |
| Desensitization | 0.81 (0.29–2.31) | 0.696 |
| For-cause biopsy | 0.68 (0.25–1.90) | 0.467 |
| TPL–Bx interval, per month | 1.01 (1.00–1.02) | 0.296 |
| Serum creatinine, per mg/dL | 1.26 (0.68–2.31) | 0.462 |
| eGFR at Bx, per mL/min/1.73 m² | 1.01 (0.98–1.03) | 0.559 |
| Sum of sDSA, per 1000 MFI | 1.23 (0.99–1.53) | 0.061 |
| Highest sDSA, per 1000 MFI | 1.22 (0.98–1.52) | 0.069 |
| sDSA C1q binding^*^ | 6.58 (0.14–299.4) | 0.308 |
| **Multivariable analysis (n = 60)** | | |
| Age | 0.99 (0.95–1.03) | 0.494 |
| Sum of sDSA, per 1000 MFI | 1.23 (0.98–1.53) | 0.070 |
| TPL–Bx interval, per month | 1.01 (1.00–1.02) | 0.429 |

The multivariable model included three covariates with *P* value < 0.4 in univariable analysis (age, sum of sDSA, and TPL–Bx interval) (The highest sDSA was excluded from the model due to multicollinearity with the sum of sDSA). ^*^sDSA C1q-binding assay results were available in only 23 patients and were selectively ordered at physician discretion, limiting robust analysis. Due to complete separation in the C1q subgroup analysis, Firth penalized logistic regression was applied.

*Abbreviations*: gDSA, intragraft donor-specific antibodies; sDSA, serum donor-specific antibodies; OR, odds ratio; CI, confidence interval; TPL, transplantation; Bx, biopsy; eGFR, estimated glomerular filtration rate; MFI, mean fluorescence intensity.

Definition of “Sum of sDSA, per 1000 MFI”: The odds ratio for this variable represents the multiplicative change in odds of gDSA positivity per 1000-unit increase in cumulative sum of sDSA MFI.

# SUPPLEMENTARY FIGURE LEGENDS

**SUPPLEMENTARY FIGURE 1. Distribution** of the highest MFI of (A) serum DSA (sDSA) and (B) graft DSA (gDSA) according to histologic findings in 60 graft biopsies. DSA-negative biopsies were imputed as 50 (half of the MFI 100 cutoff). Box plots display median and interquartile range with whiskers extending to 1.5×IQR; outliers are shown as open circles. **P <* 0.05, ***P <* 0.01 by Mann–Whitney U test with Bonferroni correction for six pairwise comparisons versus the no-rejection group.

**SUPPLEMENTARY FIGURE 2.** Distribution of (A) the MFI sum of graft DSA adjusted for biopsy length and (B) the MFI sum of graft DESA (donor HLA epitope-specific antibodies) according to histologic findings in 60 graft biopsies. The adjusted graft DSA MFI sum was calculated as raw graft DSA MFI sum × (mean biopsy length / individual biopsy length). Donor HLA epitope-specific antibodies comprised serologic gDSA together with serologically non-DSA anti-HLA antibodies sharing epitopes with donor HLA antigens (determined by epitope analysis). Box plots display median and interquartile range with whiskers extending to 1.5×IQR; outliers are shown as open circles. **P <* 0.05, ***P <* 0.01 by Mann–Whitney *U* test with Bonferroni correction for six pairwise comparisons versus the no-rejection group.

# SUPPLEMENTARY FIGURES


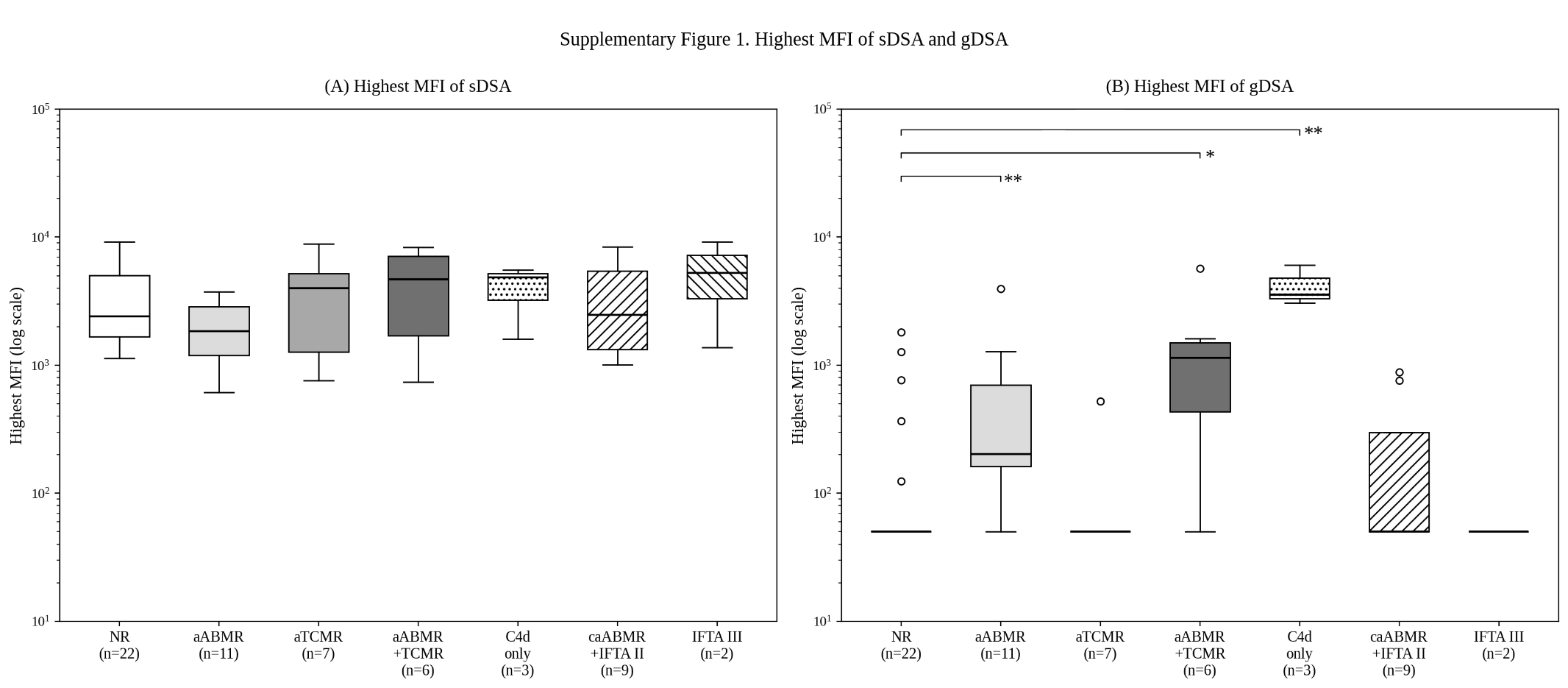


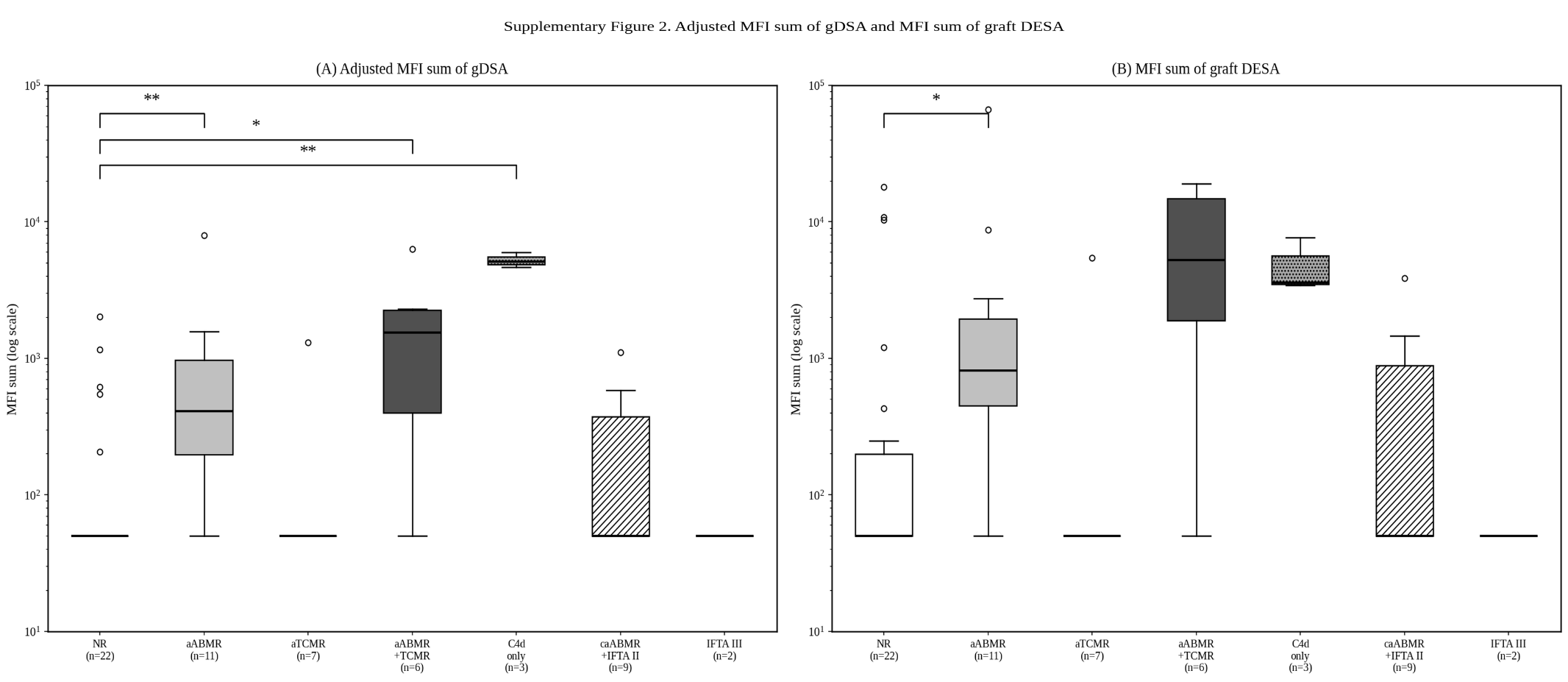

Supplement: Supplementary file 1 [file Table1.docx]
